# Involvement of *TP53* in osteosarcoma - challenges and prospects

**DOI:** 10.3389/fonc.2025.1605080

**Published:** 2025-11-13

**Authors:** Yue Shen, Shuzhou Huang, Geng Chen, Guangda Wang, Laijian Sui

**Affiliations:** 1 Binzhou Medical University, Yantai, Shandong, China; 2 Shandong Second Medical University, Weifang, Shandong, China; 3 Yantai Yuhuangding Hospital, Yantai, Shandong, China

**Keywords:** *TP53*, p53, mutation, osteosarcoma, molecular mechanism

## Abstract

Osteosarcoma (OS), the most common primary malignant bone tumor, remains a therapeutic challenge because of its high metastatic potential, chemoresistance, and poor prognosis. Mutations in the *TP53* tumor suppressor gene, including loss-of-function (LOF) and gain-of-function (GOF) mutations, play a central role in OS progression by disrupting cell cycle regulation, DNA repair, and apoptosis and promoting immune evasion and metabolic reprogramming. This review provides an in-depth analysis of p53 biology in OS, highlighting its impact on therapeutic resistance and tumor progression. We discuss advancements in radiotherapy, chemotherapy, and immunotherapy, emphasizing strategies targeting mutant *TP53* and its associated pathways. Emerging approaches, including metabolic reprogramming, noncoding RNA regulation, and precision biomarkers such as miRNAs and histone modifications, offer promising tools for diagnosis, risk stratification, and treatment optimization. By linking the molecular mechanisms of p53 with novel therapeutic strategies, this review underscores opportunities for translational research aimed at improving the clinical outcomes of OS patients.

## 1 Introduction

Osteosarcoma is a malignant tumor arising from mesenchymal tissues and displays a bimodal age distribution: the first peak occurs between ages 10 and 14, whereas the second peak appears after age 60 (1). The pathogenesis of osteosarcoma primarily involves mutations or inactivation of tumor suppressor genes, particularly *TP53*, and overexpression of oncogenes such as MDM2 (mouse double minute 2 protein) (2). The *TP53* gene, located on chromosome 17 (17p13.1), encodes a transcription factor that regulates key cellular processes, including cell cycle arrest, DNA repair, and apoptosis.

Under normal physiological conditions, the negative regulator MDM2 ubiquitinates the C-terminal lysine residues of p53, promoting its proteasomal degradation and maintaining low intracellular p53 levels. However, during carcinogenesis, The p53 ubiquitination is inhibited, leading to its accumulation and hyperactivation. Posttranslational modifications such as phosphorylation and acetylation stabilize p53 and increase its transcriptional activity, thereby modulating downstream signaling pathways (3).

Notably, the majority of human cancers harbor *TP53* mutations, predominantly missense mutations within the DNA-binding domain. These mutations are categorized into structural mutations, which disrupt protein folding, and contact mutations, which alter interactions with DNA. More than half of *TP53* mutations exhibit gain-of-function (GOF) properties, enabling oncogenic activities such as drug resistance and immune evasion. In osteosarcoma, these mutant p53 proteins not only lose their tumor-suppressive abilities but also acquire functions that actively promote tumor progression (4).

Like mutant p53, wild-type p53 is also regulated by MDM2. Elevated levels of MDM2 confer an adaptive advantage against chemotherapy by persistently suppressing wild-type p53, which prevents p53-mediated cell cycle arrest in response to DNA-damaging agents, ultimately facilitating chemoresistance (5).

This review consolidates current knowledge on the molecular mechanisms of p53 in osteosarcoma, highlighting its role in tumorigenesis and therapeutic resistance. Furthermore, we propose novel strategies for targeting p53 and its regulatory pathways to inform future research and clinical applications in osteosarcoma treatment.

## 2 Type and role of p53 in osteosarcoma

Since its discovery in 1979, p53 has been the subject of extensive and detailed research. In 1993, mutant p53 was first confirmed to promote tumor progression through a gain-of-function (GOF) mechanism. Subsequent studies have demonstrated its multifaceted roles in regulating tumor cell motility, genomic instability, differentiation and stemness, metabolic reprogramming, the tumor microenvironment, immune responses, and resistance to cancer therapies (4).

### 2.1 Wild-type p53

The wild-type p53 protein is maintained at low steady-state levels through continuous ubiquitination by the MDM2 E3 ligase, followed by proteasomal degradation (3, 6). This tightly controlled degradation ensures cellular homeostasis. However, most missense mutations in *TP53* occur in its core domain, an intrinsically unstable region characterized by low thermodynamic and kinetic stability. This inherent instability enables rapid transitions between folded and unfolded states, which contributes to the antitumor properties of wtp53 and its sensitivity to conventional chemotherapy and radiotherapy (7).

### 2.2 Mutant-type p53

The majority of *TP53* mutations in OS are missense mutations within the DNA-binding domain. These mutations not only impair the tumor-suppressive functions of wtp53 but also endow mutp53 with oncogenic properties independent of wtp53 activity. The most commonly mutated codons in TP53—R175, R248, and R273—are broadly classified into structural mutations and contact mutations, depending on the integrity of the protein’s conformation (8).

#### 2.2.1 Structural mutation

Comprehensive genomic and transcriptomic analyses of 148 osteosarcoma patients revealed that structural variations, loss of coding regions, while the promoter region is preserved and repositioned, frequently occur in the TP53 gene (9). This rearrangement often generates recurrent driver mutations by fusing the *TP53* promoter with oncogenic genes, enabling tumor cells to bypass DNA damage checkpoints and evade surveillance mechanisms (10). Notably, in younger patients, where somatic mutation accumulation is limited, these structural alterations—such as enhancer repositioning or copy number changes affecting TP53 intron 1—compensate for the deficit (11). Although such strategies can activate unrelated genes, they also highlight the complexity of TP53 regulation (9). Notably, introns 5 and 7 exhibit relatively high break frequencies, as reported by the International Cancer Genome Consortium (ICGC), suggesting that selective advantages are conferred by these breaks. Conversely, introns 2, 3, and 8 remain intact across all tumor samples, underscoring a molecular basis for this preferential selection (12). Future efforts to precisely identify ectopic reconnection points will be crucial for understanding the oncogenic potential of these mutations.

#### 2.2.2 Contact mutation

Contact mutations alter the DNA-binding residues of p53 while preserving the protein’s conformation. For example, the p53R270H mutation in female mice results in significantly greater GOF activity than structural mutants such as p53R172H (13). Moreover, BACH1 (BTB domain and CNC homologue 1) interacts with mutant p53R175H, forming a complex with SLC7A11 (xCT) and p53 that accelerates ferroptosis pathways (14). In mouse models, the R270C mutation (corresponding to human R273C) replaces the wild-type allele with a mutant allele, thereby suppressing residual wtp53 function through a dominant-negative mechanism, resulting in loss of heterozygosity. Despite these oncogenic properties, the absence of R270C does not impair metastatic progression, indicating limited therapeutic value for targeting this mutation alone (15). Recent studies have identified a dominant subclone in patients with recurrent osteosarcoma with a novel *TP53*-KPNA3 translocation and deletion of the wild-type *TP53* allele, suggesting its prognostic significance (16).

The inefficacy of the MAP (methotrexate, adriamycin, cisplatin) chemotherapy regimen in certain osteosarcoma patients is often linked to mutp53-mediated chemoresistance. For example, a 2023 study utilizing multimodal-targeted next-generation sequencing (mmNGS) revealed the instability of the homozygous variant *TP53* rs1642785 in U2OS/MTX-resistant cells. Additionally, the study identified significant allelic variations in SLC19A1 (solute carrier family 19 member 1) and rs1051266, as well as fusion transcripts of DHFR (ex4) and MSH3 (ex9). Together, these findings provide foundational insights into methotrexate resistance mechanisms (17).

In a pig model of early-onset osteosarcoma, a p2-driven mutant subtype, R167H-D152p53, was shown to impair the expression of CTR1 (copper transporter 1, SLC31A1) by preventing the transcription factor SP1 from translocating to the nucleus. This disruption leads to cisplatin resistance (18). Interestingly, the zinc finger and glutamine domains of SP1 act as copper sensors, suggesting its therapeutic potential for copper-induced cell death in osteosarcoma. Furthermore, frameshift mutants such as I332fs*14 predominantly exist as monomers, retaining the N-terminal domain that binds MDM2 and inhibits *TP53* transcription. Compared with wtp53, this mutant has significantly reduced clonal migration ability and similar drug sensitivity, suggesting that p53 oligomerization is a promising novel therapeutic strategy (6).

## 3 Degradable and modification of p53

### 3.1 Degradation of p53 in osteosarcoma

#### 3.1.1 The core role of MDM2 in p53 degradation

MDM2 antagonizes p53 via two principal mechanisms: it suppresses *TP53*’s transactivating structural domain and facilitates the ubiquitin-dependent degradation of p53-chromatin interactions (19). Interestingly, murine embryos deficient in MDM2 are embryonically lethal, a condition that can be rescued by simultaneous p53 deletion, underscoring the critical balance between these two proteins (13). In osteosarcoma, mutant p53 (mutp53) forms a complex with heat shock protein 90 (HSP90), thereby inhibiting MDM2 and CHIP (carboxy-terminus of Hsp70-interacting protein) E3 ligase activity. This interaction stabilizes mutp53, allowing it to engage in pro-oncogenic processes. The HSP90 inhibitor analogue 17-AAG (17-allylamino-17-demethoxygeldanamycin) disrupts the HSP90-mutp53 complex, reactivating endogenous MDM2 and CHIP activity, which promotes mutp53 degradation and exerts tumor-suppressive effects (20).

In addition to these canonical pathways, RBM10, an RNA-binding motif protein, enhances p53 stability by disrupting the MDM2–p53 feedback loop and inhibiting ubiquitination (21). Recent therapeutic advances have identified novel MDM2 inhibitors, such as RG-7388 and Nutlin-3, which induce apoptosis in SJSA-1 osteosarcoma cells through complementary mechanisms (22). Additionally, VIP116, a scaffold-binding peptide that targets MDM2-P53 interactions, has demonstrated promising potential as a therapeutic intervention (23).

Emerging evidence highlights the role of miR-15a in regulating p53 degradation. When delivered via serum-derived exosomes, miR-15a is internalized by osteosarcoma (OS) cells, where it directly targets GATA-binding protein 2 (GATA2), inhibiting transcription and binding to the MDM2 promoter. This reduces p53 degradation, suggesting that optimizing exosome-mediated delivery of miR-15a could serve as a novel strategy to develop p53 agonists (24).

Notably, while affecting tumor cell proliferation by suppressing p53, MDM2 also promotes tumor immune escape by regulating immune cell function (25). In the osteosarcoma microenvironment, MDM2 overexpression can inhibit the survival and effector function of CD8^+^ T cells, leading to the reduced the activity of tumor-infiltrating lymphocytes (TILs), and promoted macrophage polarization toward the M2 phenotype, thereby enhancing the immunosuppressive microenvironment. Clinical data indicate that MDM2 expression levels correlate positively with immune checkpoint molecules such as PD-L1, suggesting it may influence patient prognosis by modulating the immune microenvironment (26, 27). Further studies revealed that M2 macrophages secrete factors such as IL-10 and TGF-β to suppress T-cell antitumor activity and express immune checkpoints like PD-1 and CD47, further weaken the immune response. Targeting MDM2 reverses macrophage polarization states. For instance, combining MDM2 inhibitors with CSF1R inhibitors reduces M2 macrophage infiltration and improves the immune microenvironment in osteosarcoma (28–30). Additionally, by reducing the expression of NK cell activation receptor ligands (such as NKG2D and DNAM-1) on tumor cell surfaces, MDM2 suppresses p53 function, which diminishes NK cell-mediated osteosarcoma cells eradication. Whilst using MDM2 inhibitors (e.g., Nutlin-3a) to restore p53 activity, upregulates ligand expression and enhances NK cell-mediated tumor lysis were observed. Preclinical studies demonstrate that combining MDM2 inhibitors with adoptive NK cell therapy significantly suppresses osteosarcoma growth (31, 32).

#### 3.1.2 The synergistic role of MDM4 in p53 degradation

In contrast to MDM2, MDM4 lacks intrinsic E3 ubiquitin ligase activity but forms heterodimers with MDM2 to increase p53 degradation (33). Small-molecule inhibitors, such as bicyclic β-amino acids (Abh-AAs), effectively disrupt p53-MDM2 and p53-MDM4 interactions. Among these, tAbh-AA, characterized by an all-trans amide bond and a left-handed extended helix structure, has shown promise as an intracellular protein–protein interaction (PPI) modulator owing to its hydrophobicity and low molecular weight (34). Similarly, spiropyrazoline oxindoles, dual inhibitors of PPIs, have emerged as potential anticancer agents (35).

#### 3.1.3 Other degradation pathways of p53

In addition to the MDM2 family, RFWD2 (ring finger and WD domain 2), also known as COP1 (constitutive photomorphogenic 1), acts as an E3 ubiquitin ligase for p53. In HOS (p53mut/-) and U2OS (p53wt/wt) cells, RFWD2-mediated p53 degradation influences osteosarcoma progression (36). Verteporfin (VP), an FDA-approved drug with autophagy-modulating properties, inhibits autophagic processes and disrupts autophagosome–lysosome fusion in OS cells, leading to the formation of high-molecular-weight p53 aggregates. These aggregates impair cellular proteostasis, and their effects are amplified when they are combined with MG-132, a proteasome inhibitor that routes p53 to the lysosome (37). Overall, modulating autophagy and protein homeostasis represents a promising therapeutic avenue for osteosarcoma treatment.

### 3.2 Modification of p53 in osteosarcoma

Posttranscriptional modifications of p53 are critical to its function and stability. Forkhead box P1 (FOXP1), a transcription factor in the forkhead family, directly interacts with p53 to inhibit its transcriptional activation within the nucleus (38). Among these modifications, phosphorylation plays a central role. For example, chitooligosaccharide (COS), a drug carrier, significantly enhances p53 phosphorylation in the p53/mTOR pathway, thereby promoting autophagy and apoptosis in osteosarcoma cells (39). Similarly, in the p53/Myc pathway, disrupting the Runx consensus site mR1 in the Myc promoter or impairing Runx3 reduces Myc expression, effectively decreasing tumorigenicity in p53-deficient osteosarcoma cells. RUNX3 coactivates p53 by regulating DNA damage-induced phosphorylation at Ser15, increasing p53 stability and promoting apoptosis (40).

Phosphorylation is complemented by acetylation. p53 is a substrate for wild-type p53-induced phosphatase 1 (WIP1), which inhibits its phosphorylation at Ser15 and acetylation at Lys382. Interestingly, WIP1 also regulates p53 acetylation by modulating its interaction with p300, an acetyltransferase (40). DBC1 (deleted in breast cancer gene 1), a substrate for WIP1, indirectly affects p53 acetylation, although depletion of DBC1 does not disrupt WIP1-mediated suppression. Actinomycin D (ActD) further modulates this pathway by inhibiting SIRT1, an NAD+-dependent deacetylase, resulting in increased p53 acetylation. This triggers the upregulation of proapoptotic proteins such as NOXA and BAX and increases the expression of the antiproliferative protein p21, ultimately inducing cell cycle arrest (41). A summary of the major molecular mechanisms of p53 dysregulation discussed in this chapter and their corresponding therapeutic strategies is provided in **Table 1**.

**Table 1:** Major molecular mechanisms of p53 dysregulation in osteosarcoma and corresponding therapeutic strategies.

Molecular mechanism	Therapeutic strategy	Key agents/targets	Reference
MDM2-mediated p53 degradation	MDM2 inhibitors	RG-7388, Nutlin-3, VIP116, miR-15a	(22–24)
MDM4-enhanced p53 degradation	Dual MDM2/MDM4 inhibitors	Abh-AAs, spiropyrazoline oxindoles	(34, 35)
Mutant p53 stabilization by HSP90	HSP90 inhibitors	17-AAG	(20)
Autophagy-mediated p53 aggregation	Autophagy modulation	Verteporfin (VP), MG-132	(37)
p53 mutation-induced radioresistance	Radiosensitizers	Pep7-PSAASPV, APE1/ATM inhibitors	(42, 43)
p53 mutation-induced chemoresistance	ABCB1/P-gp inhibitors, SP1 modulation	—	(44, 45)
Mutant p53 GOF promoting immune evasion	Immunotherapy combinations	MDM2 inhibitors + anti-PD-1/PD-L1, OBP-702 + anti-CTLA-4	(46–50)
p53-mediated metabolic reprogramming	Glycolysis inhibitors	Pramlintide, GLUT1 inhibitors	(51)
p53 loss promoting stemness	ERα-targeted therapy, SKP2 inhibition	—	(36, 37)
p53 mitochondrial apoptosis pathway dysfunction	Apoptosis inducers	Panax notoginseng saponins (PNS)	(52)

## 4 The p53-mediated osteosarcoma treatment approach

**Figure 1:**
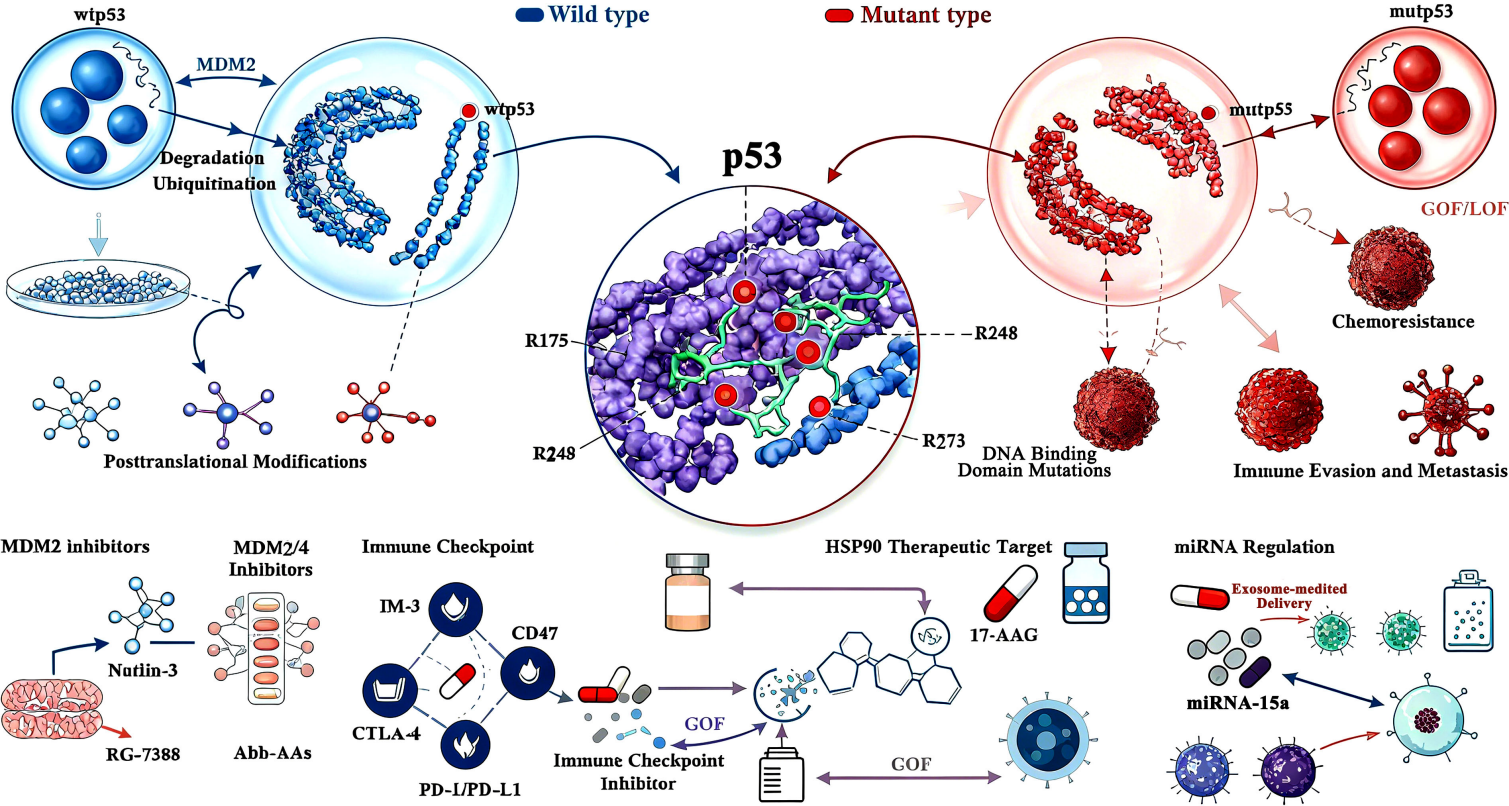
Schematic diagram of p53 (wtp53/mutp53) molecular mechanisms and therapeutic targets in osteosarcoma. This image is a schematic diagram illustrating the molecular mechanisms of p53 (encoded by the TP53 gene) in osteosarcoma (OS) and the corresponding therapeutic strategies, grounded in the core content of the document Involvement of P53 in Osteosarcoma - Challenges and Prospects. Centrally, the diagram differentiates between two key forms of p53: wild-type p53 (wtp53) and mutant p53 (mutp53). For wtp53, it highlights its regulation by MDM2, depicting MDM2’s role in mediating wtp53 ubiquitination and proteasomal degradation— a process that maintains low intracellular wtp53 levels under normal physiological conditions, but is disrupted during OS development to allow wtp53 accumulation or dysfunction. For mutp53, the diagram categorizes its mutations into functional types: Gain-of-Function (GOF) and Loss-of-Function (LOF), as the document emphasizes that over 50% of OS cases harbor TP53 mutations with these dual functional impacts, which drive tumor progression, chemoresistance, and immune evasion. The diagram also details critical posttranslational modifications (PTMs) of p53, specifically phosphorylation and acetylation— two key modifications highlighted in the document that stabilize p53 and enhance its transcriptional activity, thereby regulating downstream pathways like cell cycle arrest and apoptosis. Additionally, it identifies p53 DNA Binding Domain Mutations (a major mutational hotspot in OS, per the document) and links specific mutations (e.g., R175, R248, R273) to pathogenic effects. Therapeutically, the diagram maps targeted strategies aligned with the document’s focus: MDM2 inhibitors (e.g., Nutlin-3, RG-7388), dual MDM2/MDM4 inhibitors (e.g., Abh-AAs), HSP90 inhibitors (e.g., 17-AAG) that disrupt mutp53 stabilization, immune checkpoint inhibitors (e.g., anti-PD-1/PD-L1, anti-CTLA-4) to reverse mutp53-mediated immune evasion, and exosome-mediated delivery systems (e.g., for miR-15a) that modulate p53 activity. It also references biomarkers like miRNA signatures (e.g., miR-15a, miR-34a) and immune-related targets (e.g., CD47 on tumor-associated macrophages) that the document identifies as critical for OS diagnosis, prognosis, and treatment stratification. Overall, the diagram synthesizes the document’s key insights, visually connecting p53’s molecular behavior in OS to translational therapeutic approaches.

While surgical advancements, including ablation techniques, have shifted from focusing solely on survival to preserving limb functionality, and innovations in bone tissue engineering and material science have improved the repair and reconstruction of bone and soft tissue defects (1), patients often experience a decline in quality of life postsurgery. Moreover, postoperative recurrence rates remain alarmingly high (53). As previously highlighted, mutant p53 (mutp53) plays a central role in osteosarcoma (OS) progression, with no evident correlation to clinical factors (54). Current therapeutic strategies target either the restoration of wild-type p53 tumor suppressor functions or the inhibition of mutp53 oncogenic activities. Secondary approaches focus on disrupting critical downstream pathways and interactions of mutp53 to suppress its gain-of-function (GOF) effects (4) As shown in **Figure 1** and **Table 2**.

**Table 2:** Potential biomarkers for diagnosis, prognosis, and treatment stratification in osteosarcoma.

Biomarker type	Biomarker	Clinical utility	Reference
Genetic Alterations	TP53 structural variants	Prognosis, therapeutic targeting	(9, 12)
	TP53-KPNA3 fusion	Prognostic significance in recurrence	(16)
miRNA Signatures	miR-34a, miR-192, miR-215	Risk stratification, prognosis	(55)
	miR-539	Early diagnosis, targets TRIAP1	(56)
Histone Modifications	H4K20me3 status, SUV420H2	Early detection	(57)
Immune Microenvironment	TYROBP, TLR4, ITGAM	Predict ICI sensitivity, immune stratification	(26, 49)
	CD8^+^ T cell infiltration	Prognostic marker for OS and PFS	(58)
lncRNA/miRNA Axes	LINC-PINT, GAS5, SNHG15	Therapeutic targets, chemosensitivity predictors	(59–62)
Metabolic Markers	GLUT1 expression	Indicator of glycolytic activity, target for metabolic therapy	(18)
Macrophage Polarization	M2 macrophage markers	Immunosuppressive microenvironment indicator	(28–30)

### 4.1 Radiotherapy

Osteosarcoma (OS) is notably resistant to ionizing radiation (IR), which poses a significant challenge for effective treatment. The peptide Pep7-PSAASPV, a 7-amino acid fragment, competes with the RNA-binding protein RBM38 for binding to eukaryotic translation initiation factor 4E (eIF4E) on p53 mRNA. This high-affinity interaction allows Pep7-PSAASPV to disrupt the binding of RBM38, which inhibits p53 mRNA translation. Consequently, this action promotes p53 expression and may help overcome the radiation resistance observed in osteosarcoma cells (42). In the context of the IR-induced DNA damage response, ataxia-telangiectasia mutated (ATM) functions as a key initiator that mediates radioresistance in cancer. APE1/Ref-1 (apurinic/apyrimidinic endonuclease-reduction/oxidation factor 1) is a multifunctional protein involved in DNA repair and redox activities. Upon exposure to IR, osteosarcoma cells upregulate APE1 expression, which activates ATM through its redox activity. This activation leads to a marked reduction in p53 expression, thereby enhancing the radiation resistance of tumor cells. As a potential therapeutic strategy, the combined use of an APE1 redox inhibitor and an ATM inhibitor may effectively sensitize OS cells to IR (43).

### 4.2 Chemotherapy

In terms of chemotherapy, incomplete expression of p53 is notably associated with a reduced response to DNA-damaging agents, representing a primary mechanism of drug resistance (63). Multiple mechanisms of multidrug resistance (MDR) have been identified in osteosarcoma, including the overexpression of drug efflux pumps, decreased drug uptake, enhanced DNA damage response (DDR), dysregulation of apoptosis, and epithelial–mesenchymal transition (44). Notably, p53 does not play a role in apoptosis induced by severe DNA damage, protein turnover dysfunction, or spindle misassembly in osteosarcoma cells (64, 65). Recent findings from a 2023 study involving SaOS-2_DoxR (doxorubicin-resistant SaOS-2 subline) indicated that gain-of-function mutations in p53 can increase the expression of the ATP-binding cassette (ABC) family member ABCB1, leading to increased P-glycoprotein expression. This protein facilitates ATP-dependent drug efflux without impacting cellular permeability (44, 45).

### 4.3 Combination immunotherapy

Immunotherapy is another promising avenue, particularly with the role of immunogenic cell death (ICD) in releasing damage-associated molecular patterns (DAMPs), such as ATP and HMGB1. Compared with chemotherapy, ICD preferentially induces ATP release, activating antitumor immune responses. Given the aforementioned immunoregulatory role of MDM2, the immunosuppressive microenvironment of osteosarcoma limits the efficacy of monotherapy with immune checkpoint inhibitors (ICI). A combination strategy is required: MDM2 inhibitors (such as RG7388) restore p53 function and enhance tumor antigen presentation, while concurrent administration with anti-PD-1/PD-L1 inhibitors can release T-cell suppression, synergistically inducing antitumor immunity (46, 47). Targeting immune checkpoints on tumor-associated macrophages (TAMs) such as PD-1 and CD47, or employing Colony Stimulating Factor 1 Receptor (CSF1R) inhibitors, can reduce M2 macrophage infiltration and enhance T cell function (48, 49). The p53-armed telomerase-specific oncolytic adenovirus OBP-702 enhanced the infiltration of cytotoxic CD8+ T cells and induced systemic effects on untreated tumors, outperforming naked OBP-301, which combination with anti-CTLA-4 (Cytotoxic T-Lymphocyte-Associated protein 4) produces distant antitumor effects (50). The presence of CD8+ T cells significantly impacts overall survival (OS) and progression-free survival (PFS), with particularly strong effects noted in male patients. Additionally, a notable correlation exists between tumor-infiltrating CD4+ T cells and CD44 expression in tumor samples, suggesting that these infiltrating T cells provide protective effects for OS patients. Analyzing tumor-infiltrating lymphocytes (TILs) and associated tumor markers may aid in stratifying patients and monitoring therapeutic responses, ultimately facilitating the development of improved immunotherapy strategies to increase the efficacy of cytotoxic TILs in targeting tumor cells (58).

It is worth noting that, the p53 shift mutant I332fs*14 retains some antiproliferative capacity and displays exclusive nuclear localization, making it a candidate for targeted therapies (6). Estrogen receptor alpha (ERα)-targeted therapies show promise in augmenting existing chemotherapies for p53-positive osteosarcoma (36). Additionally, SKP2 (S-phase kinase-associated protein 2), which encodes a substrate recognition factor for the SCF E3 ubiquitin ligase, has been implicated in the immune microenvironment. In TKO (Rb1-/-; p53-/-; SKP2-/-) tumors, increased expression of immune microenvironment-infiltrating genes was observed, suggesting that SKP2 may facilitate immune rejection of OS tumors and promote antitumor immunity (66). Furthermore, the mutational status of TP53 significantly influences clinical responses in canine osteosarcoma, as missense TP53 mutations and low pretreatment blood monocyte counts correlate with longer disease-free intervals (DFIs). Patients with extended DFIs also exhibit increased transcript levels of genes related to antitumor immune responses, indicating that these factors should be considered in the development of alternative therapeutic strategies for human OS (67). Finally, the long noncoding RNA PURPL, which is induced by CD14+ peripheral blood mononuclear cells to form tumor-associated macrophage-like cells in MG-63 osteosarcoma cells, plays a regulatory role in p53 expression and may facilitate tumor development (68).

### 4.4 Other therapeutic prospects

#### 4.4.1 Application of a mitotic catastrophe

While low levels of chromosomal instability (CIN) can promote tumor development, paclitaxel (PTX) exerts its anticancer effects by inducing spindle misassembly (SM), resulting in CIN that exceeds the maximum tolerance threshold within tumor cells. Importantly, this elevated CIN-mediated tumor suppression occurs independently of p53, and the combination of the Bcl-2 inhibitor ABT-263 with paclitaxel merely accelerates the transition of cells into the late apoptotic phase. Interestingly, p53 heterozygous females exhibit a shorter tumor latency and reduced survival than their male counterparts do, a trend also observed in human malignancies, even when analyses exclude sex-differentiated tumors (64, 69). This raises the question of whether sex-specific factors, often overlooked, should be considered in the development of new drug therapies.

Furthermore, RanGAP1 is expressed at low levels in human osteosarcoma (OS) and recurrent pancancer. In a RanGAP1 knockout mouse model, aneuploidy was observed, with some chromosomes displaying tetraploid characteristics typical of high-grade OS. This resulted in hyperactivation of the spindle assembly checkpoint (SAC), amplifying the divisive chr1q chromosome, which contains MDM4, leading to p53 degradation and inactivation of the DNA damage checkpoint (DDC), ultimately enabling tumor escape (13). In these mice, deletion of RanGAP1 induced chromosome missegregation, specifically affecting chr1q and chr14q, which inhibited the expression of crucial genes involved in key signaling pathways involved in skeletal development, such as TGF-β/BMP and PI3K/AKT. Consequently, this dysregulation disrupts skeletal development (70). Thus, exploring the upregulation of RanGAP1 or its enhancers may provide a novel perspective in the treatment of osteosarcoma.

#### 4.4.2 Screening of disease development markers

TRIAP1 (TP53-regulated inhibitor of apoptosis 1) is a key target of miR-539 and is significantly upregulated by p53 in response to low levels of genotoxic stress. It interacts with Hsp70 to inhibit the formation of the Apaf-1/procaspase-9 complex, demonstrating a notable inhibitory effect on osteosarcoma cells (56). Future research may focus on the potential of miR-539 as a tool for early tumor diagnosis. Additionally, modifications by SUV420H2 (lysine methyltransferase 5C) and the trimethylation status of histone H4 at lysine 20 (H4K20me3), which are implicated in several pathways, including mitogen-activated protein kinase and p53 signaling, have been proposed as candidate biomarkers for the early detection of osteosarcoma (57). Furthermore, recent studies indicate that miR-34a, miR-192, and miR-215 may serve as prognostic markers for risk stratification in osteosarcoma (55). Risk scoring models based on immune-related genes, such as TYRO protein tyrosine kinase-binding protein (TYROBP), Toll-Like Receptor 4 (TLR4) and Integrin Subunit Alpha M (ITGAM) can predict sensitivity to immune checkpoint inhibitors (ICIs) in osteosarcoma patients. Patients in the low-risk group exhibit high infiltration of tumor-associated macrophages (TAMs) and high expression of immune checkpoint molecules, along with significantly improved prognosis. This suggests that immune microenvironment characteristics may serve as biomarkers for treatment stratification (26, 49).

#### 4.4.3 miRNA–mRNA functional axes

TP53 is situated at the heart of a complex molecular regulatory network that orchestrates cell cycle arrest and apoptosis by modulating the transcription of various genes, including microRNAs (miRNAs/miRs) (71). For example, miR-125b targets the MDM2 inhibitor p14ARF, whereas miR-34c, a transcriptional target of p53, plays a role in downregulating Notch1 (16, 72, 73). Additionally, p53 inhibits its own transcription by targeting the promoter region of miR-181b, a member of the miR-181 family, which is known to activate Wnt signaling (commonly referred to as the Wnt/beta-catenin signaling pathway). Correspondingly, miR-181b can bind to the 3’-UTR (untranslated region) of TP53, thereby suppressing p53 expression; this reciprocal regulatory mechanism establishes a negative feedback loop that governs the proliferation and invasive capabilities of osteosarcoma (OS) cells (71). Similarly, miR-203, which is also involved in regulating the Wnt pathway, indirectly modulates this pathway by increasing the level of DKK-1 (Dickkopf Wnt signaling pathway inhibitor 1) (72).

LINC-PINT (long intragenic noncoding RNA p53-induced transcript) suppresses cancer cell proliferation, invasion, and migration in osteosarcoma by downregulating miRNA-21 (59). GAS5 (growth arrest-specific transcript 5), a long noncoding RNA (lncRNA) with a stable circular structure, acts as a sponge for miR-26b-5p, increasing the expression of its target gene TP53INP1 (tumor protein p53-induced nuclear protein 1) and thereby increasing the sensitivity of osteosarcoma cells to cisplatin (DDP) through the GAS5/miR-26b-5p/TP53INP1 axis (60). Similarly, p53 binds to the -2000– to -1500-bp region of SNHG15 (small nucleolar RNA host gene 15), which leads to a reduction in SNHG15 expression and the sponging of miR-335-3p, resulting in the upregulation of ZNF32 (zinc finger protein 32). Through this mechanism, p53 downregulates SNHG15 expression in OS, and SNHG15 further inhibits cisplatin-induced apoptosis and reactive oxygen species (ROS) accumulation via the miR-335-3p/ZNF32 pathway (61). Moreover, the overexpressed lncRNA NR_027471 functions as a sponge for miR-8055, impacting TP53INP1 levels and consequently inhibiting the proliferation and progression of osteosarcoma cells (62).

#### 4.4.4 Metabolic reprogramming

In addition, an FDA-approved medication for type 2 diabetes has been shown to inhibit tumor growth in osteosarcoma cells that express full-length homologous isoforms of p53 (TAp63 and TAp73) by upregulating islet amyloid polypeptide (IAPP)-regulated metabolic programming (51). Furthermore, p53-regulated SP1 activates the GLUT1 promoter, which plays a critical role in regulating aerobic glycolysis in osteosarcoma and consequently promotes carcinogenesis (18). Given the high glycolytic capacity (GC) of osteosarcoma cells, the ability of PramLide to enhance IAPP-regulated metabolic programming provides a promising avenue for inhibiting tumor growth. This metabolic intervention highlights the potential for further research into glycolytic modulation as a therapeutic strategy in osteosarcoma.

#### 4.4.5 Stem cell differentiation

Induced pluripotent stem cells (iPSCs) have emerged as promising models for studying disease (74). Skeletal stem cells (SSCs) residing in the endoskeletal region of the bone marrow are now recognized for their ability to efficiently generate osteosarcoma (OS) and serve as potent progenitor cells, particularly under conditions of p53 deficiency (75). Notably, fibroblast growth factor receptor 3-positive (Fgfr3+) endosteal stromal cells develop aggressive OS-like lesions following the loss of p53 (76).

#### 4.4.6 Mitochondrial pathway of apoptosis

Panax notoginseng saponins (PNS) have been shown to activate the p53 mitochondrial pathway, resulting in a dose-dependent increase in the opening of the mitochondrial permeability transition pore (MPTP) and a concomitant reduction in the mitochondrial membrane potential (MMP). p53 influences the mitochondrial pathway by regulating the protein expression of Bcl-2 and Bax. This regulation can lead to a decreased MMP, increased MPTP opening, and subsequent mitochondrial dysfunction. Consequently, the release of cytochrome c into the cytoplasm activates Apaf-1, which then triggers caspase 9 and caspase 3, initiating the apoptotic pathway (52).

## 5 Conclusion

The pivotal role of p53 dysregulation in osteosarcoma pathogenesis and therapeutic resistance is now well-established. Mutant p53 proteins, particularly those with gain-of-function mutations, contribute significantly to disease progression and are present in over 50% of osteosarcoma cases. These mutations are categorized into structural and contact types based on their distinct mechanisms of disrupting p53 function, with structural mutations frequently involving non-random intronic breakpoints that may confer selective advantages during tumor evolution. The precise characterization of these genetic alterations provides not only insights into tumor biology but also critical opportunities for clinical translation.

From a diagnostic perspective, the recurrent identification of specific mutant alleles in patients experiencing sequential relapses—as well as in experimentally validated drug-resistant models—offers a strong rationale for developing mutation-specific prognostic biomarkers and targeted therapeutic strategies. These findings are particularly relevant for overcoming methotrexate resistance, a major clinical challenge in osteosarcoma management. The translation of these molecular insights into clinically applicable tools represents a promising direction for personalized treatment approaches.

Therapeutically, significant progress has been made in developing agents that target p53 pathways, including novel MDM2 inhibitors and p53-stabilizing compounds, several of which are currently in preclinical and early clinical development. Beyond conventional chemotherapy, contemporary research emphasizes combinatorial strategies that address resistance mechanisms through immunomodulation, metabolic targeting, and stem cell pathway inhibition. Emerging approaches such as mutation-specific promoter editing, enhancer reprogramming, and functional genetic screens offer additional avenues for identifying therapeutic vulnerabilities. Collectively, these advances are shaping a new paradigm of precision medicine in osteosarcoma, providing hope for improved outcomes through biologically rational and individualized treatment strategies.

## Glossary

OS: Osteosarcoma
TP53: Tumor Protein P53
LOF: Loss-of-Function
GOF: Gain-of-Function
MDM2: Mouse Double Minute 2 Homolog
miRNA/miR: MicroRNA
HSP90: Heat Shock Protein 90
CHIP: Carboxy-terminus of Hsp70-Interacting Protein
17-AAG: 17-Allylamino-17-demethoxygeldanamycin
RBM10: RNA Binding Motif Protein 10
eIF4E: Eukaryotic Translation Initiation Factor 4E
ATM: Ataxia Telangiectasia Mutated
APE1/Ref-1: Apurinic/Apyrimidinic Endonuclease 1/Redox Factor 1
DDR: DNA Damage Response
ABCB1: ATP Binding Cassette Subfamily B Member 1
P-gp: P-glycoprotein
ICD: Immunogenic Cell Death
DAMPs: Damage-Associated Molecular Patterns
HMGB1: High Mobility Group Box 1
ICI: Immune Checkpoint Inhibitor
PD-1: Programmed Cell Death Protein 1
PD-L1: Programmed Death-Ligand 1
CTLA-4: Cytotoxic T-Lymphocyte-Associated Protein 4
TILs: Tumor-Infiltrating Lymphocytes
ERα: Estrogen Receptor Alpha
SKP2: S-Phase Kinase-Associated Protein 2
DFI: Disease-Free Interval
lncRNA: Long Non-Coding RNA
CIN: Chromosomal Instability
PTX: Paclitaxel
SM: Spindle Misassembly
SAC: Spindle Assembly Checkpoint
DDC: DNA Damage Checkpoint
TRIAP1: TP53 Regulated Inhibitor of Apoptosis 1
H4K20me3: Histone H4 Lysine 20 Trimethylation
TYROBP: TYRO Protein Tyrosine Kinase Binding Protein
TLR4: Toll-Like Receptor 4
ITGAM: Integrin Subunit Alpha M
GAS5: Growth Arrest-Specific Transcript 5
TP53INP1: Tumor Protein P53 Induced Nuclear Protein 1
SNHG15: Small Nucleolar RNA Host Gene 15
ZNF32: Zinc Finger Protein 32
ROS: Reactive Oxygen Species
IAPP: Islet Amyloid Polypeptide
GLUT1: Glucose Transporter 1
GC: Glycolytic Capacity
iPSCs: Induced Pluripotent Stem Cells
SSCs: Skeletal Stem Cells
Fgfr3: Fibroblast Growth Factor Receptor 3
PNS: Panax Notoginseng Saponins
MPTP: Mitochondrial Permeability Transition Pore
MMP: Mitochondrial Membrane Potential

## References

[1] Zhao X Wu Q Gong X Liu J Ma Y . Osteosarcoma: A review of current and future therapeutic approaches. BioMed Eng Online. (2021) 20:24. doi: 10.1186/s12938-021-00860-0, PMID: 33653371 PMC7923306

[2] Nirala BK Yamamichi T Yustein JT . Deciphering the signaling mechanisms of osteosarcoma tumorigenesis. Int J Mol Sci. (2023) 24. doi: 10.3390/ijms241411367, PMID: 37511127 PMC10379831

[3] Wang H Guo M Wei H Chen Y . Targeting P53 pathways: mechanisms, structures, and advances in therapy. Signal Transduct Target Ther. (2023) 8:92. doi: 10.1038/s41392-023-01347-1, PMID: 36859359 PMC9977964

[4] Zhang C Liu J Xu D Zhang T Hu W Feng Z . Gain-of-function mutant P53 in cancer progression and therapy. J Mol Cell Biol. (2020) 12:674–87. doi: 10.1093/jmcb/mjaa040, PMID: 32722796 PMC7749743

[5] Kiri S Ryba T . Cancer, metastasis, and the epigenome. Mol Cancer. (2024) 23:154. doi: 10.1186/s12943-024-02069-w, PMID: 39095874 PMC11295362

[6] Tong DR Zhou W Katz C Regunath K Venkatesh D Ihuegbu C . P53 frameshift mutations couple loss-of-function with unique neomorphic activities. Mol Cancer Res. (2021) 19:1522–33. doi: 10.1158/1541-7786.Mcr-20-0691, PMID: 34045312 PMC8419077

[7] Zhang S Carlsen L Hernandez Borrero L Seyhan AA Tian X El-Deiry WS . Advanced strategies for therapeutic targeting of wild-type and mutant P53 in cancer. Biomolecules. (2022) 12. doi: 10.3390/biom12040548, PMID: 35454137 PMC9029346

[8] Thoenen E Curl A Iwakuma T . Tp53 in bone and soft tissue sarcomas. Pharmacol Ther. (2019) 202:149–64. doi: 10.1016/j.pharmthera.2019.06.010, PMID: 31276706 PMC6746598

[9] Saba KH Difilippo V Kovac M Cornmark L Magnusson L Nilsson J . Disruption of the tp53 locus in osteosarcoma leads to tp53 promoter gene fusions and restoration of parts of the Tp53 signalling pathway. J Pathol. (2024) 262:147–60. doi: 10.1002/path.6219, PMID: 38010733

[10] Herold N . A guardian turned rogue: tp53 promoter translocations rewire stress responses to oncogenic effectors in osteosarcoma. Cancer Gene Ther. (2024) 31:805–6. doi: 10.1038/s41417-024-00749-9, PMID: 38409586 PMC11192626

[11] Ribi S Baumhoer D Lee K Edison Teo AS Madan B . Tp53 intron 1 hotspot rearrangements are specific to sporadic osteosarcoma and can cause li-fraumeni syndrome. Oncotarget. (2015) 6:7727–40. doi: 10.18632/oncotarget.3115, PMID: 25762628 PMC4480712

[12] Beird HC Lin D Lazar AJ Futreal PA . Patterns of structural variants within tp53 introns and relocation of the tp53 promoter: A commentary(†). J Pathol. (2024) 263:131–4. doi: 10.1002/path.6270, PMID: 38482738

[13] Xiong S ChaChad D Zhang Y Gencel-Augusto J Sirito M Pant V . Differential gain-of-function activity of three P53 hotspot mutants *in vivo*. Cancer Res. (2022) 82:1926–36. doi: 10.1158/0008-5472.Can-21-3376, PMID: 35320355 PMC9117479

[14] Wang L Pan S . The regulatory effects of P53 on the typical and atypical ferroptosis in the pathogenesis of osteosarcoma: A systematic review. Front Genet. (2023) 14:1154299. doi: 10.3389/fgene.2023.1154299, PMID: 37065475 PMC10090352

[15] Shimizu T Sugihara E Takeshima H Nobusue H Yamaguchi R Yamaguchi-Iwai S . Depletion of R270c mutant P53 in osteosarcoma attenuates cell growth but does not prevent invasion and metastasis *in vivo*. Cells. (2022) 11. doi: 10.3390/cells11223614, PMID: 36429043 PMC9688353

[16] de Azevedo JWV de Medeiros Fernandes TAA Fernandes JV Jr. de Azevedo JCV Lanza DCF Bezerra CM . Biology and pathogenesis of human osteosarcoma. Oncol Lett. (2020) 19:1099–116. doi: 10.3892/ol.2019.11229, PMID: 31966039 PMC6955653

[17] Casotti C Hattinger CM Patrizio MP Luppi S Fantoni L Pasello M . Single-nucleotide polymorphism profiling by multimodal-targeted next-generation sequencing in methotrexate-resistant and -sensitive human osteosarcoma cell lines. Front Pharmacol. (2023) 14:1294873. doi: 10.3389/fphar.2023.1294873, PMID: 38074116 PMC10698553

[18] Yong L Shi Y Wu HL Dong QY Guo J Hu LS . P53 inhibits ctr1-mediated cisplatin absorption by suppressing sp1 nuclear translocation in osteosarcoma. Front Oncol. (2022) 12:1047194. doi: 10.3389/fonc.2022.1047194, PMID: 36776364 PMC9910081

[19] Henningsen KM Manzini V Magerhans A Gerber S Dobbelstein M . Mdm2-driven ubiquitination rapidly removes P53 from its cognate promoters. Biomolecules. (2021) 12. doi: 10.3390/biom12010022, PMID: 35053170 PMC8773640

[20] Yang J Li YH He MT Qiao JF Sang Y Cheang LH . Hsp90 regulates osteosarcoma cell apoptosis by targeting the P53/tcf-1-mediated transcriptional network. J Cell Physiol. (2020) 235:3894–904. doi: 10.1002/jcp.29283, PMID: 31595984 PMC13482711

[21] Cao Y Di X Zhang Q Li R Wang K . Rbm10 regulates tumor apoptosis, proliferation, and metastasis. Front Oncol. (2021) 11:603932. doi: 10.3389/fonc.2021.603932, PMID: 33718153 PMC7943715

[22] Natarajan U Venkatesan T Dhandayuthapani S Dondapatti P Rathinavelu A . Differential mechanisms involved in rg-7388 and nutlin-3 induced cell death in sjsa-1 osteosarcoma cells. Cell Signal. (2020) 75:109742. doi: 10.1016/j.cellsig.2020.109742, PMID: 32827690

[23] Zhou Z Zalutsky MR Chitneni SK . Stapled peptides as scaffolds for developing radiotracers for intracellular targets: preliminary evaluation of a radioiodinated mdm2-binding stapled peptide in the sjsa-1 osteosarcoma model. Bioorg Med Chem Lett. (2022) 66:128725. doi: 10.1016/j.bmcl.2022.128725, PMID: 35436588 PMC9940446

[24] Wu C Li Z Feng G Wang L Xie J Jin Y . Tumor suppressing role of serum-derived exosomal microrna-15a in osteosarcoma cells through the gata binding protein 2/murine double minute 2 axis and the P53 signaling pathway. Bioengineered. (2021) 12:8378–95. doi: 10.1080/21655979.2021.1987092, PMID: 34592889 PMC8806960

[25] Sun D Qian H Li J Xing P . Targeting mdm2 in Malignancies is a promising strategy for overcoming resistance to anticancer immunotherapy. J Biomed Sci. (2024) 31:17. doi: 10.1186/s12929-024-01004-x, PMID: 38281981 PMC10823613

[26] Liang T Chen J Xu G Zhang Z Xue J Zeng H . Tyrobp, tlr4 and itgam regulated macrophages polarization and immune checkpoints expression in osteosarcoma. Sci Rep. (2021) 11:19315. doi: 10.1038/s41598-021-98637-x, PMID: 34588497 PMC8481262

[27] Zheng J Miao F Wang Z Ma Y Lin Z Chen Y . Identification of mdm2 as a prognostic and immunotherapeutic biomarker in a comprehensive pan-cancer analysis: A promising target for breast cancer, bladder cancer and ovarian cancer immunotherapy. Life Sci. (2023) 327:121832. doi: 10.1016/j.lfs.2023.121832, PMID: 37276911

[28] Huang Q Liang X Ren T Huang Y Zhang H Yu Y . The role of tumor-associated macrophages in osteosarcoma progression - therapeutic implications. Cell Oncol (Dordr). (2021) 44:525–39. doi: 10.1007/s13402-021-00598-w, PMID: 33788151 PMC12980758

[29] Ligon JA Choi W Cojocaru G Fu W Hsiue EH Oke TF . Pathways of immune exclusion in metastatic osteosarcoma are associated with inferior patient outcomes. J Immunother Cancer. (2021) 9. doi: 10.1136/jitc-2020-001772, PMID: 34021032 PMC8144029

[30] Namgaladze D Brüne B . Pharmacological activation of P53 during human monocyte to macrophage differentiation attenuates their pro-inflammatory activation by tlr4, tlr7 and tlr8 agonists. Cancers (Basel). (2021) 13. doi: 10.3390/cancers13050958, PMID: 33668835 PMC7956237

[31] Veneziani I Infante P Ferretti E Melaiu O Battistelli C Lucarini V . Nutlin-3a enhances natural killer cell-mediated killing of neuroblastoma by restoring P53-dependent expression of ligands for nkg2d and dnam-1 receptors. Cancer Immunol Res. (2021) 9:170–83. doi: 10.1158/2326-6066.Cir-20-0313, PMID: 33303573

[32] Focaccetti C Benvenuto M Pighi C Vitelli A Napolitano F Cotugno N . Dnam-1-chimeric receptor-engineered nk cells, combined with nutlin-3a, more effectively fight neuroblastoma cells *in vitro*: A proof-of-concept study. Front Immunol. (2022) 13:886319. doi: 10.3389/fimmu.2022.886319, PMID: 35967339 PMC9367496

[33] Egorova O Lau HH McGraphery K Sheng Y . Mdm2 and mdmx ring domains play distinct roles in the regulation of P53 responses: A comparative study of mdm2 and mdmx ring domains in U2os cells. Int J Mol Sci. (2020) 21. doi: 10.3390/ijms21041309, PMID: 32075226 PMC7072982

[34] Su A Tabata Y Aoki K Sada A Ohki R Nagatoishi S . Elaboration of non-naturally occurring helical tripeptides as P53-mdm2/mdmx interaction inhibitors. Chem Pharm Bull (Tokyo). (2021) 69:681–92. doi: 10.1248/cpb.c21-00238, PMID: 33952867

[35] Espadinha M Lopes EA Marques V Amaral JD Dos Santos D Mori M . Discovery of mdm2-P53 and mdm4-P53 protein-protein interactions small molecule dual inhibitors. Eur J Med Chem. (2022) 241:114637. doi: 10.1016/j.ejmech.2022.114637, PMID: 35961068

[36] Wang JY Chen CM Chen CF Wu PK Chen WM . Suppression of estrogen receptor alpha inhibits cell proliferation, differentiation and enhances the chemosensitivity of P53-positive U2os osteosarcoma cell. Int J Mol Sci. (2021) 22. doi: 10.3390/ijms222011238, PMID: 34681897 PMC8540067

[37] Saini H Sharma H Mukherjee S Chowdhury S Chowdhury R . Verteporfin disrupts multiple steps of autophagy and regulates P53 to sensitize osteosarcoma cells. Cancer Cell Int. (2021) 21:52. doi: 10.1186/s12935-020-01720-y, PMID: 33446200 PMC7807844

[38] Li H Han X Yang S Wang Y Dong Y Tang T . Foxp1 drives osteosarcoma development by repressing P21 and rb transcription downstream of P53. Oncogene. (2021) 40:2785–802. doi: 10.1038/s41388-021-01742-4, PMID: 33716296

[39] Pan Z Cheng DD Wei XJ Li SJ Guo H Yang QC . Chitooligosaccharides inhibit tumor progression and induce autophagy through the activation of the P53/mtor pathway in osteosarcoma. Carbohydr Polym. (2021) 258:117596. doi: 10.1016/j.carbpol.2020.117596, PMID: 33593530

[40] Storchova R Burdova K Palek M Medema RH Macurek L . A novel assay for screening wip1 phosphatase substrates in nuclear extracts. FEBS J. (2021) 288:6035–51. doi: 10.1111/febs.15965, PMID: 33982878

[41] Kiss A Csikos C Regdon Z Polgár Z Virág L Hegedűs C . Nmnat1 is a survival factor in actinomycin D-induced osteosarcoma cell death. Int J Mol Sci. (2021) 22. doi: 10.3390/ijms22168869, PMID: 34445574 PMC8396190

[42] Lucchesi CA Zhang J Vasilatis DM Yip E Chen X . Optimization of eif4e-binding peptide pep8 to disrupt the rbm38-eif4e complex for induction of P53 and tumor suppression. Front Oncol. (2022) 12:893062. doi: 10.3389/fonc.2022.893062, PMID: 35574389 PMC9095979

[43] Xiao H Jiang N Zhang H Wang S Pi Q Chen H . Inhibitors of ape1 redox and atm synergistically sensitize osteosarcoma cells to ionizing radiation by inducing ferroptosis. Int Immunopharmacol. (2024) 139:112672. doi: 10.1016/j.intimp.2024.112672, PMID: 39032469

[44] Boichuk S Bikinieva F Valeeva E Dunaev P Vasileva M Kopnin P . Establishment and characterization of multi-drug resistant P53-negative osteosarcoma saos-2 subline. Diagnost (Basel). (2023) 13. doi: 10.3390/diagnostics13162646, PMID: 37627905 PMC10453552

[45] Skinner KT Palkar AM Hong AL . Genetics of abcb1 in cancer. Cancers (Basel). (2023) 15. doi: 10.3390/cancers15174236, PMID: 37686513 PMC10487083

[46] Zeng Q Zeng S Dai X Ding Y Huang C Ruan R . Mdm2 inhibitors in cancer immunotherapy: current status and perspective. Genes Dis. (2024) 11:101279. doi: 10.1016/j.gendis.2024.101279, PMID: 39263534 PMC11388719

[47] Ingelshed K Melssen MM Kannan P Chandramohan A Partridge AW Jiang L . Mdm2/mdmx inhibition by sulanemadlin synergizes with anti-programmed death 1 immunotherapy in wild-type P53 tumors. iScience. (2024) 27:109862. doi: 10.1016/j.isci.2024.109862, PMID: 38784022 PMC11112618

[48] Xiao J Xiao H Cai Y Liao J Liu J Yao L . Codelivery of anti-cd47 antibody and chlorin E6 using a dual ph-sensitive nanodrug for photodynamic immunotherapy of osteosarcoma. Oncol Res. (2024) 32:691–702. doi: 10.32604/or.2023.030767, PMID: 38560565 PMC10972781

[49] Sholevar CJ Liu NM Mukarrama T Kim J Lawrence J Canter RJ . Myeloid cells in the immunosuppressive microenvironment as immunotargets in osteosarcoma. Immunotargets Ther. (2025) 14:247–58. doi: 10.2147/itt.S485672, PMID: 40125425 PMC11930235

[50] Demiya K Tazawa H Kondo H Kure M Mochizuki Y Komatsubara T . P53-armed oncolytic virotherapy induces abscopal effect in osteosarcoma by promoting immunogenic cell death. Mol Ther Oncol. (2024) 32:200845. doi: 10.1016/j.omton.2024.200845, PMID: 39108499 PMC11300929

[51] Yang Y Peng Z Flores ER Kleinerman ES . Pramlintide: A novel therapeutic approach for osteosarcoma through metabolic reprogramming. Cancers (Basel). (2022) 14. doi: 10.3390/cancers14174310, PMID: 36077845 PMC9454976

[52] Han G Zhang Y Liu T Li J Li H . The anti-osteosarcoma effect from panax notoginseng saponins by inhibiting the G(0)/G(1) phase in the cell cycle and affecting P53-mediated autophagy and mitochondrial apoptosis. J Cancer. (2021) 12:6383–92. doi: 10.7150/jca.54602, PMID: 34659528 PMC8489146

[53] Pilavaki P Gahanbani Ardakani A Gikas P Constantinidou A . Osteosarcoma: current concepts and evolutions in management principles. J Clin Med. (2023) 12. doi: 10.3390/jcm12082785, PMID: 37109122 PMC10143544

[54] Li J Qin B Huang M Ma Y Li D Li W . Tumor-associated antigens (Taas) for the serological diagnosis of osteosarcoma. Front Immunol. (2021) 12:665106. doi: 10.3389/fimmu.2021.665106, PMID: 33995397 PMC8119874

[55] Otoukesh B Abbasi M Gorgani HO Farahini H Moghtadaei M Boddouhi B . Micrornas signatures, bioinformatics analysis of mirnas, mirna mimics and antagonists, and mirna therapeutics in osteosarcoma. Cancer Cell Int. (2020) 20:254. doi: 10.1186/s12935-020-01342-4, PMID: 32565738 PMC7302353

[56] Liu H Yang M Zhang Y Yang Z Chen Z Xie Y . The effect of mir-539 regulating triap1 on the apoptosis, proliferation, migration and invasion of osteosarcoma cells. Cancer Cell Int. (2021) 21:227. doi: 10.1186/s12935-021-01909-9, PMID: 33879126 PMC8056639

[57] Piao L Yuan X Wang L Xu X Zhuang M Li J . Loss of histone H4 lysine 20 trimethylation in osteosarcoma is associated with aberrant expression ofhistone methyltransferase suv420h2. Oncol Lett. (2020) 20:26. doi: 10.3892/ol.2020.11887, PMID: 32774499 PMC7406877

[58] Casanova JM Almeida JS Reith JD Sousa LM Fonseca R Freitas-Tavares P . Tumor-infiltrating lymphocytes and cancer markers in osteosarcoma: influence on patient survival. Cancers (Basel). (2021) 13. doi: 10.3390/cancers13236075, PMID: 34885185 PMC8656728

[59] He T Yuan C Zhao C . Long intragenic non-coding rna P53-induced transcript (Linc-pint) as a novel prognosis indicator and therapeutic target in cancer. BioMed Pharmacother. (2021) 143:112127. doi: 10.1016/j.biopha.2021.112127, PMID: 34474342

[60] Li G Yan X . Long non-coding rna gas5 promotes cisplatin-chemosensitivity of osteosarcoma cells via microrna-26b-5p/tp53inp1 axis. J Orthop Surg Res. (2023) 18:890. doi: 10.1186/s13018-023-04387-z, PMID: 37993867 PMC10666340

[61] Sun YF Wang Y Li XD Wang H . Snhg15, a P53-regulated lncrna, suppresses cisplatin-induced apoptosis and ros accumulation through the mir-335-3p/znf32 axis. Am J Cancer Res. (2022) 12:816–28., PMID: 35261804 PMC8899989

[62] Chen J Miao W Yang S Yin M Zhao J Song D . Lncrna nr_027471 functions as a cerna for mirna-8055 leading to suppression of osteosarcoma by regulating the expression of tp53inp1. Front Oncol. (2020) 10:563255. doi: 10.3389/fonc.2020.563255, PMID: 33117693 PMC7550745

[63] Butera A Amelio I . Deciphering the significance of P53 mutant proteins. Trends Cell Biol. (2024). doi: 10.1016/j.tcb.2024.06.003, PMID: 38960851

[64] Ho CJ Ko HJ Liao TS Zheng XR Chou PH Wang LT . Severe cellular stress activates apoptosis independently of P53 in osteosarcoma. Cell Death Discov. (2021) 7:275. doi: 10.1038/s41420-021-00658-y, PMID: 34608124 PMC8490387

[65] Wang YC Wang LT Hung TI Hong YR Chen CH Ho CJ . Severe cellular stress drives apoptosis through a dual control mechanism independently of P53. Cell Death Discov. (2022) 8:282. doi: 10.1038/s41420-022-01078-2, PMID: 35680784 PMC9184497

[66] Ferrena A Wang J Zhang R Karadal-Ferrena B Al-Hardan W Singh S . Skp2 knockout in rb1/P53-deficient mouse models of osteosarcoma induces immune infiltration and drives a transcriptional program with a favorable prognosis. Mol Cancer Ther. (2024) 23:223–34. doi: 10.1158/1535-7163.Mct-23-0173, PMID: 37871911 PMC10842346

[67] Das S Idate R Regan DP Fowles JS Lana SE Thamm DH . Immune pathways and tp53 missense mutations are associated with longer survival in canine osteosarcoma. Commun Biol. (2021) 4:1178. doi: 10.1038/s42003-021-02683-0, PMID: 34635775 PMC8505454

[68] He F Ding G Jiang W Fan X Zhu L . Effect of tumor-associated macrophages on lncrna purpl/mir-363/pdzd2 axis in osteosarcoma cells. Cell Death Discov. (2021) 7:307. doi: 10.1038/s41420-021-00700-z, PMID: 34686652 PMC8536668

[69] Funk LC Wan J Ryan SD Kaur C Sullivan R Roopra A . P53 is not required for high cin to induce tumor suppression. Mol Cancer Res. (2021) 19:112–23. doi: 10.1158/1541-7786.Mcr-20-0488, PMID: 32948674 PMC7810023

[70] Huang M Chen B Chen X Liu T Liang S Hu H . Rangap1 maintains chromosome stability in limb bud mesenchymal cells during bone development. Cell Signal. (2024) 120:111222. doi: 10.1016/j.cellsig.2024.111222, PMID: 38729327

[71] Wan J Long F Zhang C Liu Y . Mir−181b−P53 negative feedback axis regulates osteosarcoma cell proliferation and invasion. Int J Mol Med. (2020) 45:1803–13. doi: 10.3892/ijmm.2020.4558, PMID: 32236583 PMC7169658

[72] Jacques C Tesfaye R Lavaud M Georges S Baud’huin M Lamoureux F . Implication of the P53-related mir-34c, -125b, and -203 in the osteoblastic differentiation and the Malignant transformation of bone sarcomas. Cells. (2020) 9. doi: 10.3390/cells9040810, PMID: 32230926 PMC7226610

[73] Bae Y Zeng HC Chen YT Ketkar S Munivez E Yu Z . Mirna-34c suppresses osteosarcoma progression *in vivo* by targeting notch and E2f. JBMR Plus. (2022) 6:e10623. doi: 10.1002/jbm4.10623, PMID: 35509638 PMC9059472

[74] Pang LK Pena M Zhao R Lee DF . Modeling of osteosarcoma with induced pluripotent stem cells. Stem Cell Res. (2020) 49:102006. doi: 10.1016/j.scr.2020.102006, PMID: 33022533

[75] Otani S Ohnuma M Ito K Matsushita Y . Cellular dynamics of distinct skeletal cells and the development of osteosarcoma. Front Endocrinol (Lausanne). (2023) 14:1181204. doi: 10.3389/fendo.2023.1181204, PMID: 37229448 PMC10203529

[76] Matsushita Y Liu J Chu AKY Tsutsumi-Arai C Nagata M Arai Y . Bone marrow endosteal stem cells dictate active osteogenesis and aggressive tumorigenesis. Nat Commun. (2023) 14:2383. doi: 10.1038/s41467-023-38034-2, PMID: 37185464 PMC10130060

